# The glutathione S-transferase (*PxGST2L*) may contribute to the detoxification metabolism of chlorantraniliprole in *Plutella xylostella*(L.)

**DOI:** 10.1007/s10646-021-02431-4

**Published:** 2021-06-10

**Authors:** Fei Yin, Qingsheng Lin, Xiaoxiang Wang, Zhenyu Li, Xia Feng, Muhammad Zeeshan Shabbir

**Affiliations:** 1 Institute of Plant Protection, Guangdong Academy of Agricultural Sciences, Guangzhou, P.R. China; 2 Guangdong Provincial Key Laboratory of High Technology for Plant Protection, Guangzhou, P.R. China

**Keywords:** Plutella xylostella (L.), Chlorantraniliprole, Insecticide resistance, 2-DE, RNAi

## Abstract

The diamondback moth (*Plutella xylostella* L.), is an economic pest of cruciferous plants worldwide, which causes great economic loss to cruciferous plants production. However, the pest has developed resistance to insecticides. One of such insecticides is chlorantraniliprole. The study of the mechanisms underlying resistance is key for the effective management of resistance. In this study, a comparative proteomics approach was used to isolate and identify various proteins that differed between chlorantraniliprole-susceptible and -resistant strains of *P. xylostella*. Eleven proteins were significantly different and were successfully identified by MALDI-TOF-MS. Metabolism-related proteins accounted for the highest proportion among the eleven different proteins. The function of the *PxGST2L* protein was validated by RNAi. Knockdown of *PxGST2L* reduced the GST activity and increased the toxicity of chlorantraniliprole to the diamondback moth. The resistance ratio of diamondback moth to chlorantraniliprole was reduced from 1029 to 505. The results indicated that *PxGST2L* is partly responsible for chlorantraniliprole insecticide resistance in DBM. Our finding contributes to the understanding of the mechanism underlying resistance to chlorantraniliprole in the DBM, to develop effective resistance management tactics.

## Introduction

The diamondback moth (DBM), *Plutella xylostella* (L.) (Lepidoptera: Brassicidae) is a global pest of Brassica plants (Li et al. 2016; Sarfraz et al. 2006; Takeda et al. 2006). DBM develops resistance to insecticides very rapidly due to its physiological and biochemical characteristics. At present, DBM has developed resistance to all major classes of insecticides(Arruda et al. 2020; Charleston et al. 2005; Jing et al. 2021; Li et al. 2020; Pu et al. 2010; Wang et al. 2019; Zhao et al. 2006). The lack of effective insecticides makes it difficult to prevent and control DBM. Approximately US $4–5 billion is spent globally every year to manage DBM (Furlong et al. 2013).

Chlorantraniliprole was reported to be the most active compound against lepidopteran pests in 2009. It has a novel mode of action that acts on the ryanodine receptor (RyR) (Cordova et al. 2006; Lahm et al. 2005, 2007). Due to its low mammalian toxicity, high activity and unique mechanism of action, the pesticide has been used widely. It has been registered for use in more than 80 countries and ranked among the 5 top-selling insecticides worldwide in 2011. However, long-term use of chemical agent can increase the fitness cost and survival ability of pest, leading to a likely resurgence of this pest (Desneux et al. 2007). Likewise, *Bradysia odoriphaga* (Diptera: Apicymphaeidae) developed 76 and 43.32 fold resistance to clothianidin and chlorfenapyr insecticides after 10 generations of selection. The larvae and pupae survived longer and generation cycle increase in resistant strains (Gul et al. 2019; Ullah et al. 2020a). The clothianidin adversely affect the adult longevity and fecundity of progeny of *Aphis gossypii* Glover (Hemiptera: Aphididae) at low or sublethal concentrations (Ullah et al. 2019a). The cyantraniliprole-treated strains of *Laodelphax straatellus (Fallen)* (Hemiptera: Delphacidae) increased faster compared to control strain (Zhang et al. 2019). Acetamiprid and imidacloprid affect the vitellogenin gene (Vg) expression in melon aphid, *A. gossypii* (Ullah et al. 2019b, c). Long-term use of chlorantraniliprole affect the net reproductive rate, mean generation time and doubling time of *Harmonia axyridis* (Coleoptera: Coccinellidae), *Trichogramma brassicae* (Hymenoptera: Hypteridae) and *Apis mellifera* (L.) (Hymenoptera: Apidae) (Oliveira et al. 2019; Parsaeyan et al. 2020; Williams 2020). The low contact toxicity of chlorantraniliprole affected the survival, development and predation ability of *Coccinella septempunctata* (Coleoptera: Coccinellidae) (He et al. 2019). After a long period of heavy use of chlorantraniliprole, resistance of DBM to chlorantraniliprole was reported in South China, Thailand, the Philippines and Brazil (Ribeiro et al. 2014; Wang et al. 2010; Wang and Wu 2012). In China, this resulted in an outbreak of the pest. About US $0.77 billion is spent annually on the control of DBM and crop losses in China (Li et al. 2016).

The metabolic resistance, target-site resistance, penetration and behavioral resistance are primary resistance mechanisms in the target insects (Guedes et al. 2019; Ingham et al. 2018). Previously it was reported that cytochrome P450 monooxygenases (P450s) genes was involved in acetamiprid resistance in *A. gossypii* and chlorantraniliprole resistance in *P. xylostella* (L.) (Li et al. 2018; Ullah et al. 2020b). P450 gene regulate the insecticide resistance of mosquitoes through GPCR/Gαs/AC/cAMP-PKA signaling pathways (Li and Liu 2017, 2019). Modified acetylcholinesterase (AChE) phenotype (MACE) caused by a mutation in the ACHE gene (*ace2*) is strongly involved in the resistance of *Myzus persicae* (Sulzer) (Homoptera: Aphididae) to pirimicarb (Liu et al. 2017). Most studies on the mechanisms underlying resistance in DBM have focused on genetic and physiological changes and have reported that mutation of RYR and detoxification metabolism as the two main causes (He et al. 2012; Li et al. 2020; Wang et al. 2020). Transport protein ABCC2 participates in the resistance of DBM to avermectin and chlorfenapyr (Xu et al. 2019). The Pglycoprotein gene *pXABCB1* is closely related to diamondback moth resistance to Cry1Ac toxin (Zhou et al. 2020). Over‐expression of UDP-glycosyltransferase gene *UGT2B17* and cytochrome P450 *CYP6BG1* are reported to be involved in chlorantraniliprole resistance in *P. xylostella* (L.) (Li et al. 2018; Li et al. 2016). However, gene mutations and physiological changes are influenced by proteins. Proteomics is a powerful method for gaining insight into various physiological changes at the cellular level. It has been applied in numerous studies, including immunity, resistance mechanisms and pathology, etc. (Guo et al. 2014; Etebari et al. 2011). In this study, a comparative proteomic approach was used to identify resistance-related proteins. The different expressions of metabolism-related proteins were further validated. These results make an important contribution to better understand the mechanism underlying resistance to chlorantraniliprole in DBM.

## Materials and methods

### Insects

A susceptible *P. xylostella* strain (SS) was originally collected from vegetable fields (Guangdong Province, China) in 2002 and was reared separately in the laboratory without any insecticides exposure for fourteen years.

The pupae of chlorantraniliprole resistant DBM strain (RS) were collected from vegetable fields in Guangdong Province, China and the larva were reared on *Brassica alboglabra* L. H. Bailey in the laboratory. The laboratory conditions were maintained at 25 ± 2 °C, 65 ± 5% relative humidity (RH) and 16:8 h light: dark photoperiod.

### Leaf bioassay

The leaf dip method was used in the bioassay. Cabbage leaf discs (6.5 cm in diameter) were prepared using a hole punch. The leaves were soaked in the liquid for 10 sec and then air dried 2 h at room temperature. The leaves were placed in a Petri dish with a diameter of 6.5 °C. Ten third-instars larvae of *P.xylostella* L. were transferred to each plastic Petri dish and kept at 25 ± 2 °C, 65 ± 5% RH with a photoperiod of 16: 8 (L; D). There are four repetitions per treatment. Leaves treated with distilled water alone were used as control. After 48 h, the mortality was examined. The larvae were considered to be dead when they do not respond by touching with small brush. Bioassays which showed mortality rates >10% in the control were discarded, and the whole bioassay was repeated four times.

The data were analyzed with SPSS 19 software. The resistance ratios (RRs) were estimated at the LC 50 level as RR = LC 50 of testing strain/ LC 50 of susceptible strain.

### Protein extraction

Twenty 3-instar larvae of each strain were ground thoroughly to fine powder using a mortar and pestle in liquid nitrogen. The method for total protein extraction followed the procedure described previously (Yin et al. 2017). The powder was resuspended in 1 mL of extraction buffer (40 mmol/L DTT and 10% TCA in acetone) for 1 h at 4 °C., The samples were then centrifuged at 15,000 rpm for 30 min at 4 °C. The supernatant was discarded. The precipitate was resuspended three times in 3 volumes of ice-cold acetone for 1 h at −20 °C followed by centrifugation at 12,000 rpm for 10 min. This procedure was repeated twice. The protein was resuspended in 500 μL of lysis buffer (2 mol/L thiourea, 4% (v/v) CHAPS, 2% (v/v) Bio-lyteampholytes (pH 3–10), 7 mol/L urea and 40 mmol/L DTT) for 2 h at 4 °C followed by centrifugation at 15,000 rpm for 30 min. The supernatant was stored at −80 °C and the total protein concentration was estimated according to the manufacturer’s instructions using a Bradford protein assay kit (Generay, China) (Jin et al. 2010).

### Two dimensional gel electrophoresis (2-DE) and image analysis

A total of 1 mg protein was loaded on 17-cm Immobiline dry strips (pH 3-10, NL, Bio-Rad) with rehydration buffer (2 mol/L thiourea, 40 mmol/L DTT, 2% (v/v) CHAPS, 2% (v/v) Bio-lyteampholytes (pH 3–10), 7 mol/L urea and 0.002% bromophenol blue). The gel strips were transferred to an Ettan IPG Phor system (Bio-Rad) after active rehydration for 16 h. Isoelectric focusing was performed at 250 V for 3 h, 1000 V for 4 h, 10,000 V for 5 h, and 10,000 V to reach 8000 Vhr and held at 500 V. Then, the strips were equilibrated in equilibration buffer containing 2% (w/v) SDS, 1% (w/v) DTT, 50 mmol/L Tris-HCl, pH 8.8, 20% (v/v) glycerol, 6 mol/L urea, 0.002% (w/v) bromophenol blue and 1.5% (w/v) iodoacetamide for 15 min at room temperature. The strips were then loaded onto 12% (w/v) SDS-PAGE gels and sealed with 0.5% agarose. SDS-PAGE was performed at 80 V for 30 min followed by 200 V for 4.5 h on an Ettan DALT-6 separation unit (Bio-Rad).

When SDS-PAGE was completed, the gels were stained with Coomassie and scanned using a GS 800 scanner. The protein spots were analyzed using the PDQuest software (Bio-Rad). Statistical analysis was performed with Student’s *t* test. *P* values less than 0.05 were considered statistically significant. Only differentially expressed proteins (≥2.0-fold or ≤0.5-fold) were excised and subjected to subsequent identification by mass spectrometry (MS) (Bruker Daltonics, Germany).

### MALDI-TOF/TOF-MS/MS analysis

The differentially expressed protein spots of interest were excised. The pieces were then processed by in-gel trypsin digestion and analyzed by MALDI-TOF-MS/MS (Bu et al. 2012). The MS/MS data were searched against the expressed sequence tag (EST) and NCBI protein databases using MASCOT search engine (Matrix Science, London, UK). MASCOT score over 50 was considered to be a positive hit. The molecular functions of identified proteins were classified by the GO database (http://amigo.geneontology.org/amigo/landing).

### Quantitative real-time PCR (RT-qPCR)

To investigate the differentially expressed proteins, four proteins were selected for additional analysis. Actin served as the reference gene. Total RNA was extracted from third instar larvae of DBM using an EASYspin RNA rapid isolation kit for tissue/cells (Biomed, Beijing) according to the manufacturer’s protocol. First strand cDNA was synthesized from 1 μg of RNA using M-MLV reverse transcriptase (Takara Bio Inc, kusatsu, shiga, Japan). RT-qPCR was performed in triplicate in a Rotor-Gene thermal cycler (Bio-Rad). Each 20 μl reaction mixture contained 2 μl 0.2 μM each primer (Table 1), 10 μl 2X All-in-One qPCR Mix, 2 μl diluted cDNA and 4 μl of ddH_2_O. The relative gene expression level was calculated using the 2^-ΔΔCT^method (Lin et al. 2013).

**Table 1:** Primers used in the quantitative real-time RT-PCR analysis

Target gene	Primer name	Primer sequence (5ʹ–3ʹ)
Juvenile hormone diol kinase	J-F	GGCACGAGGCAGCGACAACACAAGC
	J-R	TGCCCGTCGTTGTTGGCGTCAG
Cu/Zn superoxide dismutase	CZ-F	GAGAGCATTGGGGGCGTCACTA
	CZ-R	GGCACCCTAACATTTATGCTGAT
Glutathione S-transferase 4 protein (PxGST2L)	G-F	CGAGTTCAAGCCCAAGACCA
	G-R	CCTTCTCATCTTCTTCGTAGTGGA
Triosephosphate isomerase	T-F	AGGATGTTGGGTCTGAGTGGGT
	T-R	TGATTTCATGGATTGTGTTCTTGTC
Imaginal disk growth factor	I-F	GTGCCCGCCATCATCAACTTCG
	I-R	GCGATGCCCACCACGATCTTAG
*β-*actin	A-F	TGGCACCACACCTTCTAC
	A-R	CATGATCTGGGTCATCTTCT

### Functional verification of glutathione S-transferase 4 protein (*PxGST2L*) by RNAi

The dsRNA of *PxGST2L* genes were synthesized according to the instructions of the T7 RioMAXTM Express RNAi System. Primers are shown in Table 2. The dsRNA (2 µg/larva) was injected into 3rd instar larvae. The expression of the *PxGST2L* genes and the activity of glutathione S-transferase (GST) were evaluated 24 h after treatment following Chen’s method (Chen and Zhang 2015). The toxicity of chlorantraniliprole to DBM was then tested using the leaf-dipping method 24 h after the dsRNA treatment (Guo et al. 2014). The tests were repeated three times, and 10 DBM were used for each replication. The control group was injected with green fluorescent protein dsRNA (ds*GFP*) (CK). Concentration-mortality data (LC_50_) were analyzed using SPSS19 (Software, IBM). Resistance ratio (RR) was calculated by the formula, RR = the resistant population LC_50_/ the susceptible population LC_50_.

**Table 2:** Primers for dsRNA synthesis

Primer sequence	Primer name
GCTGTAGACGGTTTCGTAGAC	dsPxGST2L-F
TCACAATGCCGGTGGTAAA	dsPxGST2L-R
GGATCCTAATACGACTCACTATAGGTCACAATGCCGGTGGTAAA	dsPxGST2L-T7F
GGATCCTAATACGACTCACTATAGGGCTGTAGACGGTTTCGTAGAC	dsPxGST2L-T7R
AAGGGCGAGGAGCTGTTCACCG	dsGFP-F
CAGCAGGACCATGTGATCGCGC	dsGFP-R
GGATCCTAATACGACTCACTATAGGAAGGGCGAGGAGCTGTTCACCG	dsGFP-T7F
GGATCCTAATACGACTCACTATAGGCAGCAGGACCATGTGATCGCGC	dsGFP-T7R

## Results

### Toxicity of chlorantraniliprole against *P. xylostella* L

The leaf dip bioassays showed that chlorantraniliprole was toxic to the susceptible and the resistant strains with LC _50_ value of 0.22 and 108.01 mg L^−1^. The resistant ratio is 490.95 (Table 3).

**Table 3:** Toxicity of chlorantraniliprole against different strains of *P.xylostella* L.

Control	Regression equation	LC50 (mg/L)	95% confidence interval/mg·L-1	X^2^ (df)^a^	RR^b^
Resistant population	Y = 1.595–3.244	108.01	80.11–165.61	1.246 (3)	490.95
Susceptible population	Y = 1.592x + 1.061	0.22	0.13–0.36	0.696 (3)	–

^a^Chi‐square value (X2) and 180 degrees of freedom (df)

^b^RR (resistance ratio) = the resistant population LC50/ the susceptible population LC50

### 2-DE analysis of differentially expressed proteins in the CS and CR of DBM

**Fig. 1:**
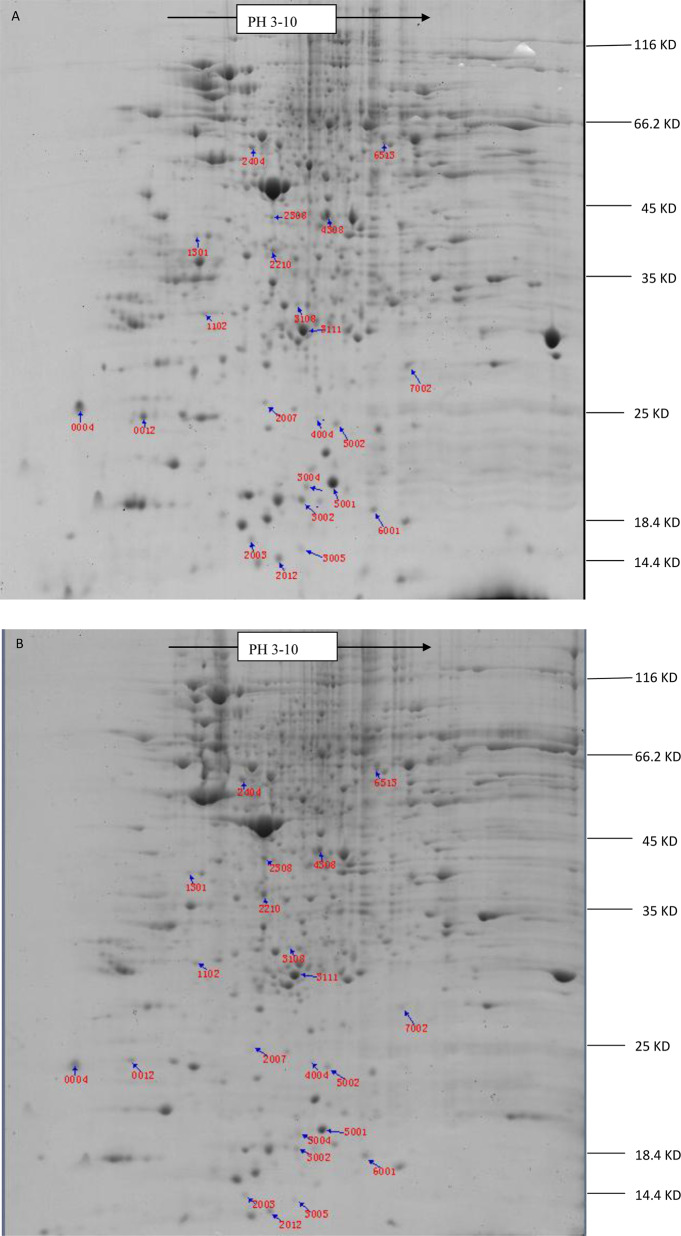
2-DE Gel analysis of the protein expression of the DBM between CS and CR. **A** resistant strain; (**B**) susceptible strain. In IEF, a total of 1 mg protein was separated on 17 cm, pH 3–10 NL immobiline dry strips. SDS-PAGE was performed with 12% gels, and the protein spots were visualized using G250 staining. 22 proteins change significantly (*P* < 0.05, fold ≥2.0). 11 proteins were identified successfully

**Fig. 2:**
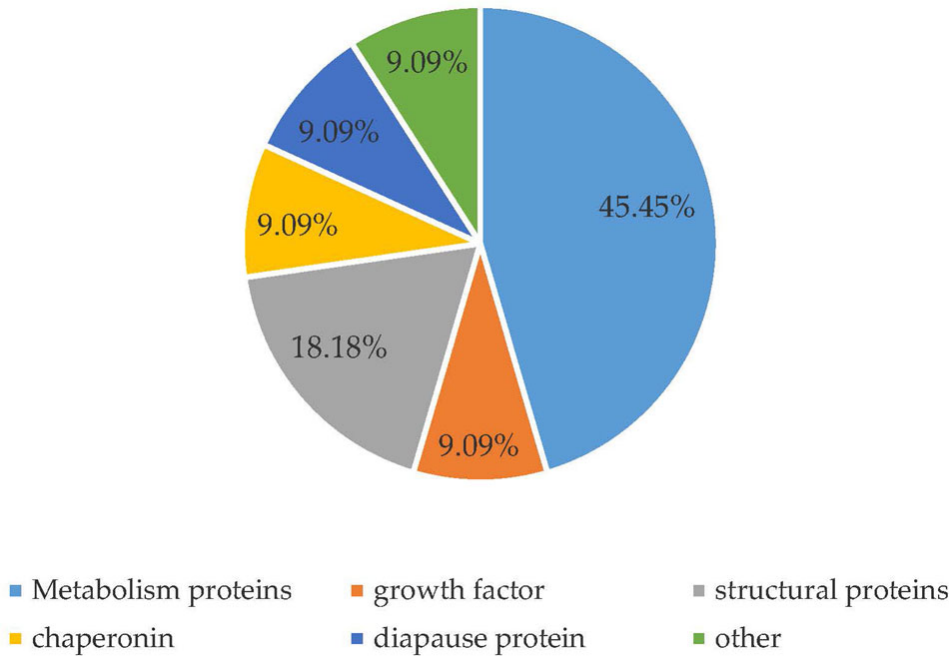
Gene ontology (Go) categories of the different proteins identified from the *P. xylostella* in CS and CR. The annotations for the identified proteins were retrieved by searching against the GO database with their sequences. The molecular functions of identified proteins were classified according to their GO annotations

The proteins were separated by 2-DE, and the gels were scanned and analyzed using the PDQuest software. Approximately 520 protein spots were detected in each gel. Twenty-two proteins were significantly different (*r* ≥ 2.00, *p* < 0.05) in abundance (Fig. 1). Eleven proteins were successfully identified by MALDI-TOF-MS/MS. All mascot scores were over 60. The expression levels of nine proteins (spots 1, 6, 7, 11, 13, 14, 15, 16 and 18) were upregulated and two proteins (spots 21 and 22) were downregulated in CR (Table 4). These proteins have important biological functions including metabolism-related, structural protein, chaperonin and diapause proteins. Metabolism-related proteins accounted for 45.45% among the 11 different proteins (Fig. 2).

**Table 4:** MALDI-TOF/TOF MS identification of differentially expressed proteins of *Plutella. Xylostella*i n CS and CR

Spot number	SSP^a^	Protein name	Accession number	Mascot score	Isoelectric point	Coverage rate (%)	Express effect^b^
1	0004	Juvenile hormone diol kinase	gi|49532884	208	4.5	24	Up
6	3002	Cu/Zn superoxide dismutase	gi|53148457	253	6.3	25	Up
7	5001	abnormal wing disc-like protein	gi|330370526	169	9.5	19	Up
11	4004	diapause bioclock protein	gi|357621212	59	6.3	7	Up
13	7002	peptidylprolyl isomerase B	gi|357609162	67	8.9	6	Up
14	3111	glutathione S-transferase 4	gi|49532926	429	5.8	31	Up
15	3108	heat shock protein 1	gi|357614091	191	6.4	23	Up
16	1102	triosephosphate isomerase	gi|440200451	201	4.7	25	Up
18	6513	imaginal disk growth factor	gi|117970190	238	8.5	13	Up
21	2210	proteasome alpha 6 subunit	gi|17737405	118	6.1	9	Down
22	2404	phosphoglycerate kinase	gi|389614755	80	5.4	11	Down

^a^SSP: protein number

^b^Up: up-regulated protein; down: down-regulated protein

### Differential gene expression at the mRNA and protein levels in CS and CR of DBM

**Fig. 3:**
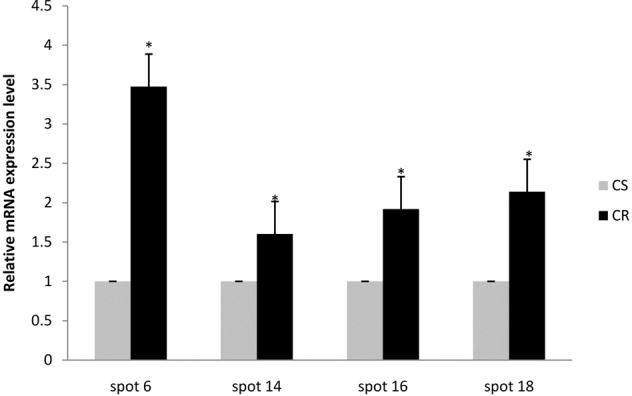
Expression of four genes at the mRNA levels between the chlorantraniliprole-sensitive (SS) and resistant (RS) strains of *P. xylostella*. Spot 6: Cu/Zn superoxide dismutase; Spot 14: glutathione S-transferase 4 protein; Spot 16: triosephosphate isomerase; Spot 18: imaginal disk growth factor. CS, susceptible *P. xylostella* strain; CR, chlorantraniliprole resistant DBM strain. The expression level of the protein and mRNA in CS was set as 1. The asterisk indicates a significant difference between the CS and CR at the 0.05 level (*t* test). “*” indicates a statistically significant difference (*P* < 0.05)

**Fig. 4:**
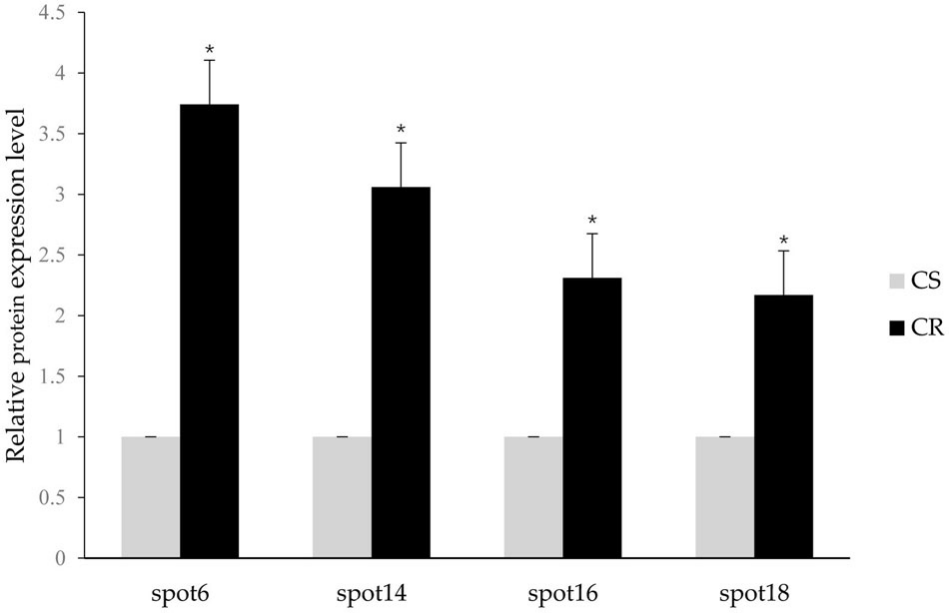
Expression of four genes at the protein levels between the chlorantraniliprole-sensitive (SS) and resistant (RS) strains of *P. xylostella*. Spot 6: Cu/Zn superoxide dismutase; Spot 14: glutathione S-transferase 4 protein; Spot 16: triosephosphate isomerase; Spot 18: imaginal disk growth factor. CS, susceptible *P. xylostella* strain; CR, chlorantraniliprole resistant DBM strain. The expression level of the protein and mRNA in CS was set as 1. The asterisk indicates a significant difference between the CS and CR at the 0.05 level (*t* test). “*” indicates a statistically significant difference (*P* < 0.05)

To investigate the mRNA expression of these proteins, four proteins (spots 6, 14, 16 and 18) were selected for additional analysis at the mRNA level using RT-qPCR. The results showed that Cu/Zn superoxide dismutase, glutathione S-transferase 4 protein, triosephosphate isomerase and imaginal disk growth factor, were significantly upregulated at the mRNA level (Fig. 3). The changes in the mRNA levels of these four proteins were consistent with the corresponding protein levels (Fig. 4).

### Functional verification of the *PxGST2L* by RNAi

**Fig. 5:**
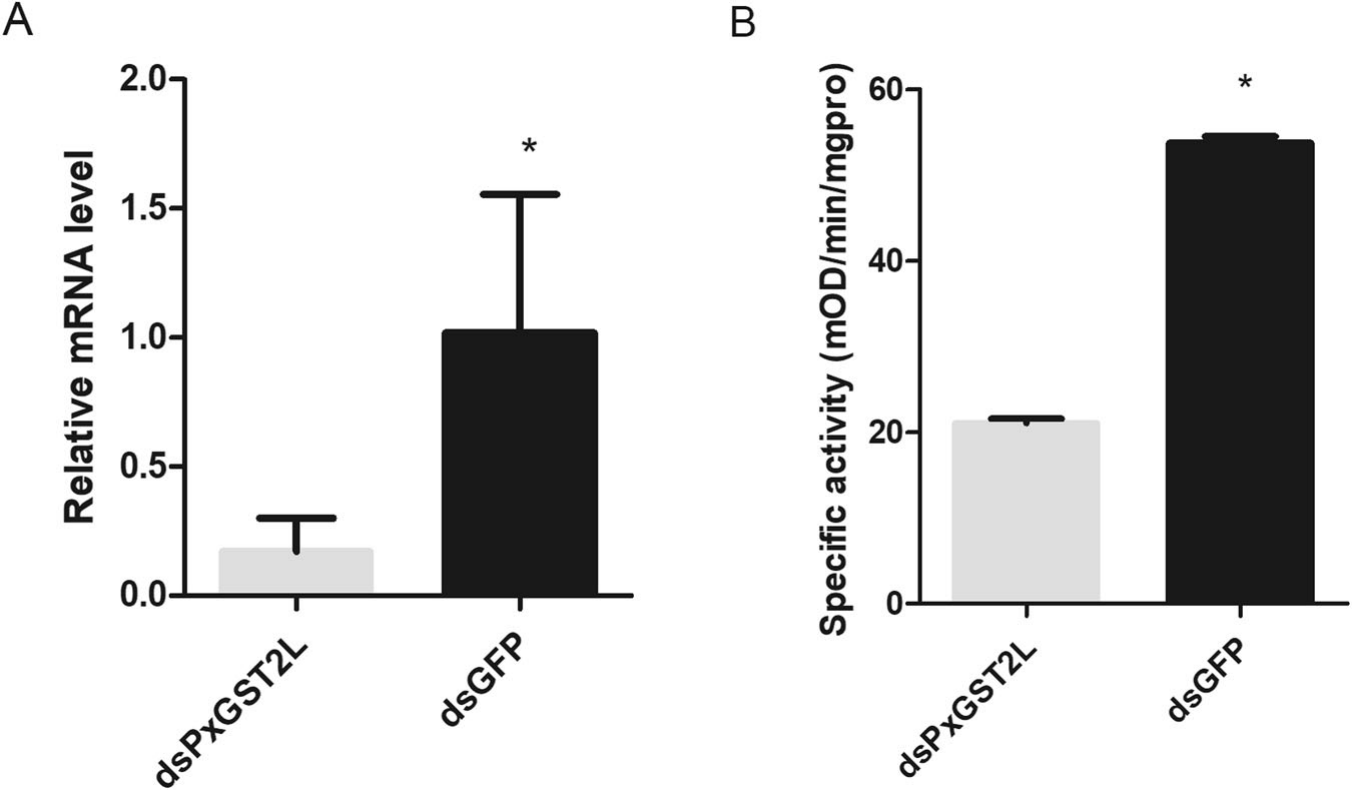
Functional analysis of *PxGST2L* by RNAi. (**A**) Relative expression of *PxGST2L* in the third-instar larvae injected with dsPx*GST2L* or ds*GFP*. (**B**) The Glutathione S-transferase activity of third-instar larvae injected with dsPx*GST2L* or ds*GFP*. Data represent the mean ± SD of three biological replicates. The asterisk * in (**A**) and (**B**) indicates a statistically significant difference (*P* < 0.05) in gene expression between control and treatment

To determine whether the highly expressed *PxGST2L* was capable of providing chlorantraniliprole resistance in the CR, it was silenced by injection of *PxGST2L* dsRNA (ds*PxGST2L*) in DBM larvae. *PxGST2L* gene expression was significantly lower than the control (ds*GFP*) 24 h after injection of dsGST2L and ds*GFP*. This indicated that the relative expression of *PxGST2L* was significantly decreased by the dsRNA treatments (2 µg /larva). The efficiency of the RNAi was >76% (Fig. 5A). The activity of GSTs was significantly decreased (more than 60%) (Fig 5B). Silencing of *PxGST2L* increased the toxicity of chlorantraniliprole to DBM. The resistance ratio of DBM to chlorantraniliprole was reduced from 1029 to 505 (Table 5).

**Table 5:** Toxicity of chlorantraniliprole against different strains of *P.xylostella* L.

Control	Regression equation	LC50 (mg/L)	95% confidence interval/mg·L-1	X^2^ (df)^a^	RR^b^
susceptible species	Y = 1.592x + 1.061	0.22	0.13–0.36	0.696 (3)	–
dsPxGST2L	Y = 0.900x − 1.860	116.22	56.94–206.21	0.479 (3)	505
dsGFP	Y = 0.692x − 1.642	236.74	118.00–1244.72	0.205 (3)	1029
CK	Y = 0.707x − 1.748	296.19	150.40–2628.15	0.084 (3)	1346

^a^Chi‐square value (X^2^) and degrees of freedom (df)

^b^RR (resistance ratio) = the resistant population LC50/ the susceptible population LC50

## Discussion

The mechanisms underlying insecticide resistance in insects are complex. Changes in protein expression can provide a basis for understanding the potential effects on pest resistance to pesticides (GuiLu and Wen 2018). Proteomics is a useful method which provides insights into mechanisms involved in physiological changes at the protein level (Zhang et al. 2019). Due to its resolution, robustness, and ability to isolate entire, intact proteins, 2-DE has become a classic method for proteomics research (Rabilloud et al. 2010). In this study, eleven differentially abundant protein spots were separated by 2-DE and were successfully identified. Nine proteins were up regulated and two proteins were down regulated in our study. Metabolism-related proteins accounted for the majority of the differentially-expressed proteins. The results provided evidence that chlorantraniliprole induces proteomic changes in *P. xylostella* and that metabolism-related proteins play an important role in the DBM’s resistance to chlorantraniliprole. This is consistent with transcriptome analysis (Lin et al. 2013).

Target mutations and the enhancement of the metabolic activities of detoxification enzymes are the two primary factors responsible for insecticide resistance in insects (Heckel 2012; Li et al. 2007; Liu et al. 2017). Different detoxification enzymes such as P450, CarE and GSTs have been reported to contribute to resistance (Li and Liu 2017, 2019; Li et al. 2018; Lumjuan et al. 2011; Ullah et al. 2020b; Wang et al. 2013). In this study, five metabolism-related differential proteins were found, and they accounted for the highest proportion of the differentially-expressed proteins. Among these, *PxGST2L* belongs to the GSTs family which is a major family of detoxification enzymes involved in phase II metabolism. The insect GSTs play key roles in the metabolism of xenobiotic compounds and can enhance insecticide resistance by binding to insecticide molecules. They are also involved in insecticide detoxification by mediating O-dealkylation and catalyzing dehydrochlorination (Chen et al. 2018; Konanz and Nauen 2004; Kostaropoulos et al. 2001; Lyall 2006; Vontas et al. 2001). The GST activity has been associated with resistance to pesticides in many insects. For example, GSTs play critical roles in chlorpyrifos detoxification in *Spodoptera litura* Fabricius (Lepidoptera:Noctuidae). Three cytosolic SlGSTs (*SlGSTe13*, *SlGSTt1* and *SlGSTz1*) and one microsomal Slgst (*SlMGST1-1*) were induced only in the resistant strain. Eight cytosolic *SlGSTs* were expressed 1.52-5.15-fold higher in the resistant strain than in the susceptible strain (Zhang et al. 2016). In *Locusta migratoria* manilensis (Orthoptera:Acridiae), *FLmGSTs3* was reported to be involved in carbaryl detoxification. After silencing the *LmGSTs3* and treating the larvae with carbaryl, the nymph mortalities increased by 38.7% (Qin et al. 2012). Further, *BdGSTe8* played a role in metabolic resistance to malathion in *Bactrocera dorsalis* (Diptera: Tephritidae). The susceptibility of a malathion-resistant strain recovered after knockdown the *BdGSTe8-B*, and overexpression of *BdGSTe8* enhanced resistance in transgenic *Drosophila*. *BdGSTe8-B* allele was also reported to be involved in the direct metabolism of malathion by metabolic assay (Lu et al. 2020). Huang et al. studied the expression characteristics of the Sigma family GSTs under pesticide stress in *Chilo suppressalis* (Walker) (Lepidoptera:Pyralidae). They found that avermectins and chlorantraniliprole induced a higher expression of the Sigma family GSTs. Their relative expression levels were increased 7.1-44.5 times under the inducement of chlorantraniliprole. The results suggested that the Sigma family GSTs plays an important role in resisting the negative effects caused by xenobiotics (Jia et al. 2011). In *P. xylostella*, the increase in GST activity enhances its resistance to organophosphates, spinosad, pyrethhroids and indoxacarb (Chen and Zhang 2015).Compared with the susceptible population, the activity of GST in the resistant population of DBM to chlorantraniliprole increased significantly. The activity of GST was twice as high as that in the susceptible population. Meanwhile, it was found that the toxicity of chlorantraniliprole to DBM can be synergized by the GSTs specific inhibitor diethy-maleate (DEM). The results indicated that GSTs play potential detoxification roles in the metabolism of DBM to chlorantraniliprole (Chen and Zhang 2015). In this study, the results of 2 - DE showed that *PxGST2L* was significantly up-regulated in CR. It showed that *PxGST2L* was closely associated with chlorantraniliprole resistance in DBM.

RNAi has been used to confirm the role of candidate detoxification genes. The genes silencing by RNAi will enhance the role of insecticides, which had been proved by the susceptibility of carbaryl to *L. migratoria* (Li et al. 2018) and the toxicity of chlorantraniliprole to DBM (Li et al. 2018). Also, the toxicity of chlorantraniliprole to DBM was increased by 26.8% from the silencing of *CYP6BG1* by RNAi. Further, the knockdown of *GSTe2* or *GSTe7* effectively increased the susceptibility of *Aedes aegypti* (Diptera:Culicidae) to deltamethrin (Li et al. 2018). The silencing efficiency recorded in this study was more than 76%. After the silencing of the *PxGST2L*, the activities of GSTs and LC_50_ of DBM to chlorantraniliprole were significantly decreased. All results provide evidence that *PxGST2L* are closely related to the resistance of DBM to chlorantraniliprole.

GST can enhance insect’s resistance by directly metabolizing or sequestrating chemicals or providing protection against oxidative stress induced by insecticide exposure. However, it was not clear in this study which mechanism caused the increased resistance to chlorantraniliprole in the DBM. It has been reported that the P450 gene is also a factor in the metabolism of DBM to chlorantraniliprole, and here GST is also a factor. But the relationship between the two metabolic enzymes needs to be further clarified. Further clarifying the regulatory metabolic pathway of GST will be helpful to the development of new drugs and the protection of diamides.

## Conclusions

In this paper, the highest proportion of metabolic proteins was found by differential proteomics analysis. The *PxGST2L* is partly responsible for chlorantraniliprole insecticide resistance in DBM. These results provide new insights into the mechanisms of resistance to chlorantraniliprole insecticides in insect pests.

## Data Availability

The authors declare the availability of data and material.
